# Involvement of atypical protein kinase C in the regulation of cardiac glucose and long-chain fatty acid uptake

**DOI:** 10.3389/fphys.2012.00361

**Published:** 2012-09-11

**Authors:** Daphna D. J. Habets, Joost J. F. P. Luiken, Margriet Ouwens, Will A. Coumans, Monique Vergouwe, Stine J. Maarbjerg, Michael Leitges, Arend Bonen, Erik A. Richter, Jan F. C. Glatz

**Affiliations:** ^1^Department of Molecular Genetics, Cardiovascular Research Institute Maastricht, Maastricht UniversityMaastricht, Netherlands; ^2^German Diabetes CenterDüsseldorf, Germany; ^3^Department of Exercise and Sport Sciences, Section of Human Physiology, Copenhagen Muscle Research Centre, University of CopenhagenCopenhagen, Denmark; ^4^Biotechnology Centre of OsloOslo, Norway; ^5^Department of Human Health and Nutritional Sciences, University of GuelphGuelph, ON, Canada

**Keywords:** cardiomyocytes, insulin, AMPK, atypical PKCs, glucose uptake, long-chain fatty acid uptake

## Abstract

**Aim:** The signaling pathways involved in the regulation of cardiac GLUT4 translocation/glucose uptake and CD36 translocation/long-chain fatty acid uptake are not fully understood. We compared in heart/muscle-specific PKC-λ knockout mice the roles of atypical PKCs (PKC-ζ and PKC-λ) in regulating cardiac glucose and fatty acid uptake. **Results:** Neither insulin-stimulated nor AMPK-mediated glucose and fatty acid uptake were inhibited upon genetic PKC-λ ablation in cardiomyocytes. In contrast, myristoylated PKC-ζ pseudosubstrate inhibited both insulin-stimulated and AMPK-mediated glucose and fatty acid uptake by >80% in both wild-type and PKC-λ-knockout cardiomyocytes. In PKC-λ knockout cardiomyocytes, PKC-ζ is the sole remaining atypical PKC isoform, and its expression level is not different from wild-type cardiomyocytes, in which it contributes to 29% and 17% of total atypical PKC expression and phosphorylation, respectively. **Conclusion:** Taken together, atypical PKCs are necessary for insulin-stimulated and AMPK-mediated glucose uptake into the heart, as well as for insulin-stimulated and AMPK-mediated fatty acid uptake. However, the residual PKC-ζ activity in PKC-λ-knockout cardiomyocytes is sufficient to allow optimal stimulation of glucose and fatty acid uptake, indicating that atypical PKCs are necessary but not rate-limiting in the regulation of cardiac substrate uptake and that PKC-λ and PKC-ζ have interchangeable functions in these processes.

## Introduction

Glucose and long-chain fatty acids are the most important cardiac substrates. Both substrates are efficiently extracted from the interstitial space by substrate specific sarcolemmal transporters. GLUT4 is well-known to be the main cardiac glucose transporter. It is also well-established that GLUT4 translocation from recycling endosomes to the plasma membrane accounts for stimulation of cardiac glucose uptake upon physiological stimuli, especially increased contractile activity and increased circulating levels of insulin (Sevilla et al., 1997; Glatz et al., 2010). With respect to fatty acid uptake, the heart expresses several distinct fatty acid transporters, most notably CD36 and members of the FATP family (FATP1 and 6) (Doege and Stahl, 2006; Glatz et al., 2010). Similar to GLUT4, in the heart also CD36 translocates from intracellular stores to the sarcolemma upon insulin and contraction stimulation (Glatz et al., 2010). Importantly, in CD36-knockout mice, stimulus-induced fatty acid uptake was completely abrogated, emphasizing a key role of CD36 in the regulation of cardiac fatty acid fluxes (Habets et al., 2007). In contrast, the quantitative importance of the FATPs in cardiac fatty acid uptake is not yet clear.

The signaling cascades involved in the regulation of GLUT4 translocation and glucose uptake have been topic of intensive research, whereas the investigation of signaling enzymes involved in the regulation of CD36 translocation and fatty acid uptake has only recently been initiated (Glatz et al., 2010). Strikingly, the signaling enzymes found to be involved in the regulation of CD36 translocation and fatty acid uptake in the heart are also known to be essential in the regulation of GLUT4 translocation and glucose uptake. Namely, the PI3K—Akt/PKB insulin signaling pathway mediates insulin-induced GLUT4 translocation as well as insulin-induced CD36 translocation in cardiomyocytes (Luiken et al., 2002, 2004). Our recent data concerning the ability of myristoylated PKC-ζ pseudosubstrate to inhibit insulin-stimulated glucose uptake and fatty acid uptake in cardiomyocytes (Luiken et al., 2009) also indicate that the atypical PKC isoform PKC-ζ is essential for insulin-induced translocation of GLUT4 and CD36, respectively. However, given that PKC-ζ was already fully active in cardiomyocytes under basal conditions and was not further activated by insulin indicated that PKC-ζ has a permissive but not a regulatory role in insulin-stimulated glucose and fatty acid uptake.

With respect to contraction-induced GLUT4 translocation as well as contraction-induced CD36 translocation in cardiomyocytes, both LKB1 and AMPKα2 have been shown to be essential (Habets et al., 2009). Interestingly, recent studies in rat (Chen et al., 2002; Sajan et al., 2010) and human (Richter et al., 2004) skeletal muscle showed that (1) treadmill exercise activated hindlimb PKC-ζ, and that (2) stimulation of GLUT4 translocation and glucose uptake by the AMPK activating agent AICAR was inhibited by myristoylated PKC-ζ pseudosubstrate, together indicating that this atypical PKC operates in the same signaling cascade as AMPK to stimulate GLUT4 translocation and glucose uptake in response to contraction. Whether PKC-ζ is also involved in AMPK-mediated CD36 translocation and fatty acid uptake in cardiomyocytes is not known yet.

In rodent muscle, two atypical PKC isoforms occur, i.e., PKC-ζ and PKC-λ. Whereas PKC-ζ is the predominant atypical PKC isoform in rat muscle tissues (Pucéat et al., 1994; Shizukuda and Buttrick, 2002), PKC-λ is the major representative of the atypical PKCs in mouse muscle tissues (Akimoto et al., 1994). Therefore, to study the role of atypical PKCs in insulin-stimulated glucose uptake into skeletal muscle, heart/muscle-specific PKC-λ knockout mice were generated (Farese et al., 2007). This indeed resulted in a large decrease in overall atypical PKC activity in muscle concomitant with a reduction of insulin-stimulated glucose uptake, confirming that atypical PKCs are essential in this process (Farese et al., 2007). These heart/muscle-specific PKC-λ knockout mice exhibited whole body insulin resistance, hyperinsulinemia, abdominal obesity, and several other lipid abnormalities, indicating that a specific defect in muscle atypical PKCs is sufficient to induce hallmark features of type-2 diabetes and the metabolic syndrome (Farese et al., 2007).

We used cardiomyocytes from these heart/muscle-specific PKC-λ knockout mice to study (1) whether PKC-λ and PKC-ζ play similar roles in insulin-stimulated glucose and fatty acid uptake into cardiomyocytes from heart/muscle-specific PKC-λ knockout mice, and to study (2) the role of atypical PKCs in AMPK-mediated glucose and fatty acid uptake.

## Materials and methods

### Materials

[1−^14^C]palmitic acid, 2-deoxy-D-[1−^3^H]glucose were from GE Healthcare (Piscataway, NJ, USA). BSA (fraction V, essentially fatty acid free), phloretin and insulin were from Sigma (St. Louis, MO). Liberase blendzyme 1 was from Roche Diagnostics (Indianapolis, IN). Antibodies against CD36 and GLUT4 were from Chemicon International Inc. (Temecula, USA). The rabbit polyclonal antibody against PKC-λ and PKC-ζ (C-20) was from Transduction Laboratories (Sparks, MD, USA). The antibody against phosphorylated acetyl-CoA carboxylase (ACC) (Ser79) was from Upstate (Dundee, UK). Anti-phospho-Akt (Ser473), anti-phospho-(Ser/Thr) Akt substrate (PAS), and anti-phospho-PKC-ζ (Thr410/403) antibodies were from Cell Signaling Technology (Beverly, MA, USA).

### Animals

Embryonic stem cells containing a floxed PKC-λ allele (with loxP sites flanking exon 110-233) were used to generate C57Bl/6 mice with germline-transmitted floxed PKC-λ. These mice were crossed with mice harboring a muscle creatine kinase-driven Cre recombinase transgene to generate heterozygous and homozygous heart/muscle-specific PKC-λ knockout mice and various littermates (Farese et al., 2007). The mice were bred in the Muscle Research Centre, University of Copenhagen, according to the guidelines of that institution. After shipment to Maastricht, experiments were performed according to the guidelines of the Experimental Animal Committee of Maastricht University.

### Isolation and pre-incubation of mouse cardiomyocytes

Mouse cardiomyocytes were isolated from hearts of male homozygous heart/muscle-specific PKC-λ knockout mice and their wild-type male littermates (mice, 6–8 months of age) using a Langendorff perfusion system as previously described (Habets et al., 2007). Cell suspensions (2.0 ml; 5–10 mg wet mass/ml; viability 60–80%) were pre-incubated in capped incubation vials either with 0.35% DMSO (Ctrl), 100 nmol/l insulin or 1 μM oligomycin for 20 min at 37°C under continuous shaking before subsequent measurements were made. Myristoylated PKC-ζ pseudosubstrate (50 μM) was added 20 min prior to addition of insulin or oligomycin.

### Protein expression and phosphorylation

Pellets of stimulated cell suspensions were dissolved in sample buffer, as previously described (Habets et al., 2007), and subjected to SDS-polyacrylamide gel electrophoresis, followed by Western blotting for the detection of GLUT4, CD36, VAMP2 and GAPDH, and of phospho-AS160 (PAS antibody), phospho-ACC (Ser79), phospho-Akt (Ser473) by applying the antibodies according to the manufacturer's instructions.

### Palmitate and deoxyglucose uptake

For palmitate and deoxyglucose uptake, 0.5 ml of a mixture of [1−^14^C]palmitate/BSA complex and [1−^3^H]deoxyglucose was added to pre-incubated cell suspensions (final concentrations: palmitate, 100 μmol/l; deoxyglucose, 100 μmol/l; palmitate/BSA ratio 0.3). The rate of uptake of ^14^C-palmitate and of ^3^H-deoxyglucose in a 3-min incubation, i.e., within the initial uptake phase, was determined as previously described (Habets et al., 2007). The uptake was terminated by adding of an ice-cold stop solution containing 0.2 mmol/l phloretin, followed by washing the cells twice for 2 min at 45 *g* and assessing the radioactivity in the pellet.

### Quantitative gene expression

Total RNA was isolated from cardiomyocytes using Tri-Reagent (Sigma). Total RNA (500 ng) was reverse transcribed using the iScript™ cDNA synthesis kit (Bio-Rad). Quantitative PCR was performed on the MyiQ Single Color Real-Time PCR Detection System (BioRad) using 10 ng cDNA, 300 nM of each primer and IQ SYBR Green Supermix (BioRad) in a total volume of 20 μl. Primer sequences of PKC-ζ are available upon request. Cyclophilin A was used as housekeeping gene.

### Data presentation and statistics

All data are presented as means ± S.E.M. for the indicated number of cardiomyocyte preparations. Statistical difference between means was analyzed by the paired Student's *t*-test. *P* ≤ 0.05 was considered significant.

## Results

### Characterization of cardiomyocytes from PKC-λ knockout mice

**Figure 1:**
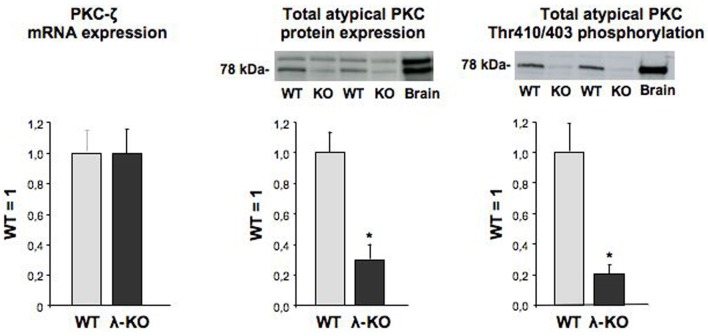
**Expression of PKC-ζ, and expression and phosphorylation of total atypical PKC in hearts from wild-type and muscle-specific PKC-λ-knockout mice.** Heart homogenates from wild-type (WT) and PKC-λ-knockout (KO) mice were used for PCR analysis of PKC-ζ (left graph) and Western detection of total atypical PKC expression and phosphorylation (middle and right graph). In case of Western detection, representative blots are displayed above each graph. Data are means ± SEM for six experiments carried out with different heart homogenates. ^*^Significantly different from WT (*P* < 0.05).

Hearts from heart/muscle-specific PKC-λ knockout mice were previously controlled for proper deletion of PKC-λ (Farese et al., 2007) using a rabbit polyclonal antibody directed against a peptide spanning residues 184-234 of the PKC-λ protein. This PKC-λ-specific antibody does not recognize PKC-ζ (Kovac et al., 2007). Indeed protein expression of PKC-λ appeared largely absent in hearts from heart/muscle-specific PKC-λ knockout mice (Farese et al., 2007). PKC-ζ expression was readily detectable in wild-type mouse heart. Moreover, its mRNA content was unchanged in PKC-λ knockout heart (Figure 1). This observation is in line with previous observations in hearts from PKC-λ knockout showing no change in protein expression levels of PKC-ζ (Farese et al., 2007). A commercailly available antibody (Santa Cruz–C20) has been commonly used to detect PKC-λ (Farese et al., 2007; Luiken et al., 2009). This rabbit polyclonal antibody recognizes the C-terminus of atypical PKCs, which is identical for PKC-λ and PKC-ζ. Therefore, this antibody cannot discriminate between each of these atypical PKC isoforms (Farese et al., 2007). Using this antibody, it was observed that total atypical PKC expression was reduced by 79% (Figure 1). When using an antibody recognizing the Thr410/403 phosphomotif within the activation loop, which is also shared by both PKC-λ and PKC-ζ, total atypical PKC activation loop phosphorylation was reduced by 87% (Figure 1).

**Figure 2:**
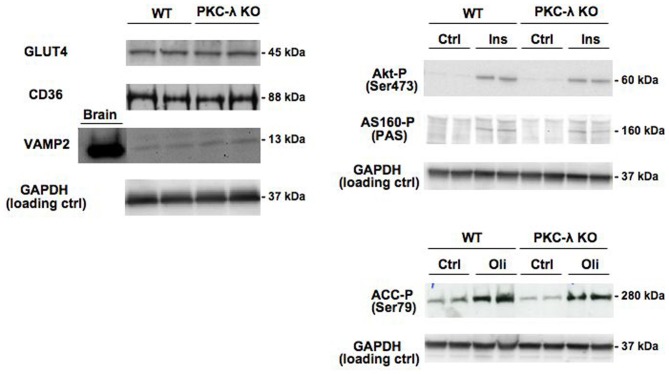
**Expression of GLUT4, CD36, and VAMP2 in cardiomyocytes from wild-type and muscle-specific PKC-λ-knockout mice, and the effects of insulin and oligomycin treatment on phosphorylation of Akt, AS160, and ACC.** Wild-type (WT) and PKC-λ-knockout (KO) cardiomyocytes were used to determine GLUT4, CD36, VAMP2, and GAPDH (loading control) expression, and after incubation for 20 min in the absence (Ctrl) or presence of 100 nM insulin (Ins) or 1 μM oligomycin (Oli) for assessment of phospho-Akt (Ser473), phospho-ACC (Ser79), and phospho-AS160 by Western blotting. In case of VAMP2, a sample from homogenized rat brain of identical protein content was used as a positive control on the expression of this protein. A representative Western blot is presented out of five experiments with different cardiomyocyte preparations.

Expression of GLUT4 and CD36 was similar in wild-type and PKC-λ knockout cardiomyocytes (Figure 2). This indicates that the potential capacity for fatty acid and glucose transport in cardiomyocytes is not altered due to heart/muscle-specific knockout of PKC-λ.

Insulin signaling through the Akt/PKB–AS160 axis was not altered due to the genetic ablation of PKC-λ in cardiomyocytes because insulin induced a similar increase in Akt/PKB-Ser473 phosphorylation and in AS160 phosphorylation at the PAS motif in wild-type and PKC-λ knockout cardiomyocytes (Figure 2). We used the mitochondrial F_1_F_0_-ATPase inhibitor oligomycin to activate AMPK signaling because oligomycin-stimulated glucose and fatty acid uptake is entirely mediated through AMPK (Habets et al., 2009). In addition to insulin signaling, AMPK signaling in cardiomyocytes was not affected by ablation of PKC-λ, because oligomycin-induced ACC-Ser79 phosphorylation was not different between wild-type and knockout cardiomyocytes (Figure 2).

VAMP2 has been reported to be a direct substrate of atypical PKCs, as shown in adipocytes (Braiman et al., 2001). Because VAMP2 is functioning as a v-SNARE in GLUT4 translocation in adipocytes (Olson et al., 1997) and muscle (Sevilla et al., 1997), an upregulation in VAMP2 expression could serve as a compensatory mechanism to maintain GLUT4 translocation. However, VAMP2 was not found to be upregulated in PKC-λ knockout cardiomyocytes (Figure 2), excluding the occurrence of this possible compensatory mechanism. Taken together, possible alterations in substrate uptake due to the ablation of PKC-λ in cardiomyocytes cannot be ascribed to compensatory changes in any of the parameters studied.

### Effect of PKC-λ ablation on glucose and fatty acid uptake

**Figure 3:**
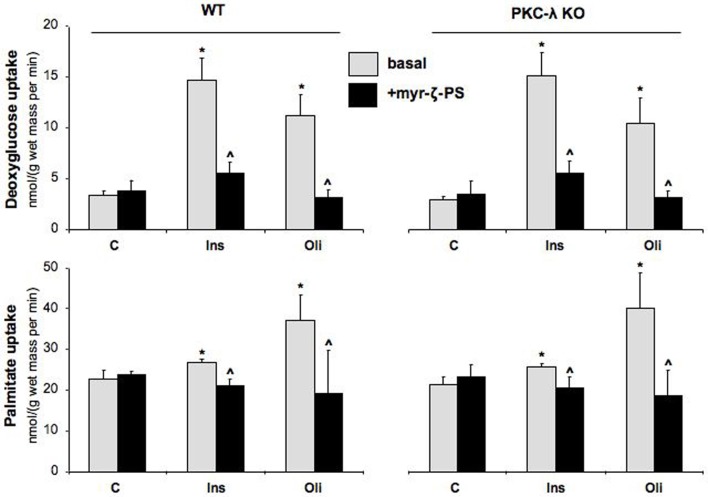
**Effects of insulin and oligomycin stimulation on deoxyglucose and palmitate uptake in cardiomyocytes from wild-type and muscle-specific PKC-λ-knockout mice.** Wild-type (WT) and PKC-λ-knockout cardiomyocytes were preincubated in the absence (basal) or presence of 50 μM myristoylated PKC-ζ pseudosubstrate (myr-ζ-PS), and subsequently incubated for 20 min in the absence (Ctrl) or presence of 100 nM insulin (Ins) or 1 μM oligomycin (Oli), whereafter uptake rates of ^3^H-deoxyglucose and ^14^C-palmitate were measured. Data are means ± SEM for six experiments carried out with different cardiomyocyte preparations. ^*^Significantly different from Ctrl (*P* < 0.05). ^'^Significantly different from corresponding basal (*P* < 0.05).

In agreement with our previous observations in wild-type cardiomyocytes (Habets et al., 2009), glucose uptake was stimulated by 4.3-fold and 3.3-fold in wild-type cardiomyocytes upon treatment with insulin or oligomycin, respectively, and fatty acid uptake by 1.2-fold and 1.6-fold (Figure 3). Surprisingly, the magnitude of stimulation of glucose and fatty acid uptake in PKC-λ knockout cardiomyocytes was comparable to that observed in wild-type cardiomyocytes (Figure 3). However, when wild-type or PKC-λ knockout cardiomyocytes were pre-incubated with myristoylated PKC-ζ pseudosubstrate, insulin- or oligomycin-stimulated glucose uptake and insulin- or oligomycin-stimulated fatty acid uptake were largely (>80%) or almost fully reduced to basal uptake levels (Figure 3). Treatment with myristoylated PKC-ζ pseudosubstrate did not affect basal fatty acid or glucose uptake (Figure 3).

## Discussion

In a recent study, we observed that in rat cardiomyocytes PKC-ζ is essential for insulin-stimulated GLUT4 translocation/glucose uptake and insulin-stimulated CD36 translocation/fatty acid uptake (Luiken et al., 2009). However, the role of PKC-ζ in these processes was proposed to be merely permissive because this atypical PKC isoform was already fully active in the heart under basal conditions and not further activated by insulin treatment. In the present study, we further examined the role of atypical PKCs in insulin-stimulated glucose and fatty acid uptake, and explored the role of atypical PKCs in AMPK-mediated glucose and fatty acid uptake in heart/muscle-specific PKC-λ knockout mice. Using cardiomyocytes from this mouse model in combination with the specific atypical PKC inhibitor myristoyted PKC-ζ pseudosubstrate, the following major observations were made: (1) atypical PKCs are essential not only for insulin-stimulated glucose and fatty acid uptake but also for AMPK-mediated glucose and fatty acid uptake, and (2) the residual atypical PKC-ζ isoform in PKC-λ knockout cardiomyocytes is sufficient to allow maximal insulin-stimulated and AMPK-mediated glucose and fatty acid uptake.

### Atypical PKCs are essential for insulin-stimulated and AMPK-mediated glucose and fatty acid uptake

Pharmacological inhibition of total atypical PKC activity, using myristoylated PKC-ζ pseudosubstrate, rather than genetic ablation of the major mouse heart atypical PKC isoform PKC-λ, revealed that atypical PKCs are necessary for both insulin-stimulated and AMPK-mediated glucose and fatty acid uptake into the heart. The myristoylated PKC-ζ pseudosubstrate is known to inhibit both PKC-ζ and PKC-λ because the sequence of this synthetic oligopeptide is present in the pseudosubstrate region of both isoforms (Akimoto et al., 1994), and is known to effectively inhibit total atypical PKC activity in cellular systems at 10–100 μM concentrations (e.g., see Standaert et al., 1997). In agreement with these findings, we have recently shown that in rat cardiomyocytes 50 μ M myristoylated PKC-ζ pseudosubstrate largely inhibited total atypical PKC activity in rat cardiomyocytes (Luiken et al., 2009). At this concentration the pseudosubstrate does not cause any inhibition of Akt phosphorylation (Luiken et al., 2009) indicating that its effect is not due to inhibition of the canonical insulin signaling pathway. Importantly, 50 μM myristoylated PKC-ζ pseudosubstrate completely inhibited insulin-stimulated glucose and fatty acid uptake into rat cardiomyocytes (Luiken et al., 2009), indicating that atypical PKCs are involved in both processes. For insulin-stimulated fatty acid uptake this was a novel finding but for insulin-stimulated glucose uptake this has been known for more than a decade, at least in adipocytes (Standaert et al., 1997).

Less research has been done to employ the myristoylated PKC-ζ pseudosubstrate in AMPK-mediated glucose uptake, and indeed this synthetic peptide revealed the involvement of atypical PKCs in this process, at least in L6 myotubes (Chen et al., 2002; Sajan et al., 2010). Hence, ours is the first study that shows that atypical PKCs are necessary for AMPK-stimulated glucose uptake into a cardiac cellular model system and for AMPK-stimulated fatty acid uptake into any cell type or tissue.

Earlier, we established in rat cardiomyocytes (Luiken et al., 2002) and in mouse cardiomyocytes (Habets et al., 2009) that enhancement of glucose uptake and fatty acid uptake upon insulin or AMPK stimulation is of similar magnitude as the increase in cell surface content of GLUT4 and of CD36, respectively, indicating that translocation of both transporters accounts for the increase in uptake of both substrates. Moreover, in the case of fatty acids, we have shown that the increase in uptake of this substrate in response to insulin stimulation (data not shown) or AMPK stimulation (Habets et al., 2009) is absent in cardiomyocytes from CD36 knockout mice, implicating the involvement of CD36 in insulin-stimulated and in AMPK-mediated fatty acid uptake. In addition, we have shown that inhibition of atypical PKCs by staurosporin completely blocks insulin-stimulated glucose and fatty acid uptake, and also insulin-stimulated GLUT4 and CD36 translocation (Luiken et al., 2009). Hence, it may be concluded that the mechanism of involvement of atypical PKCs in stimulation of glucose and fatty acid uptake encompasses the translocation of GLUT4 and CD36.

### PKC-ζ in PKC-λ knockout cardiomyocytes is sufficient for optimal stimulation of cardiac substrate uptake

Remarkably, the abilty of the myristoylated PKC-ζ pseudosubstrate to inhibit insulin-stimulated or AMPK-mediated substrate uptake into cardiomyocytes is in sharp contrast with the inability of genetic PKC-λ ablation to block these processes. The only plausible explanation to integrate these findings is to assume that in PKC-λ-knockout cardiomyocytes there is sufficient atypical PKC activity left to sustain maximal GLUT4 and CD36 translocation. Still, in the heart of heart/muscle-specific PKC-λ-knockout mice PKC-λ is largely absent (Farese et al., 2007). Importantly, our present results and those of Farese show that in PKC-λ-knockout cardiomyocytes (Figure 1) or in PKC-λ-knockout hearts (Farese et al., 2007), PKC-ζ is still present, and, moreover, the expression of this atypical PKC isoform at the mRNA level (Figure 1) or at the protein level (Farese et al., 2007) is not changed in comparison to wild-type cardiomyocytes or wild-type hearts. It is obvious that the residual total PKC expression (21%) and phosphorylation (13%) in PKC-λ-knockout cardiomyocytes (see Figure 1) can be completely ascribed to PKC-ζ. Because PKC-ζ expression is not different in wild-type and PKC-λ-knockout cardiomyocytes, it can be deduced that in these wild-type myocytes PKC-ζ presents 13–21% of total atypical PKC amount or capacity. Upon genetic deletion of PKC-λ, this remaining PKC-ζ is still sufficient for optimal regulation of substrate uptake in the heart. This implies therefore that (1) there is a redundancy/excess total atypical PKC capacity of at least 5-fold in relation to insulin-stimulated or AMPK-mediated glucose and fatty acid uptake into cardiomyocytes, so to propose that atypical PKCs are necessary but not rate-limiting in the regulation of cardiac substrate uptake, and (2) that PKC-λ and PKC-ζ can substitute for each other in the regulation of cardiac substrate uptake.

## Concluding remarks

**Figure 4:**
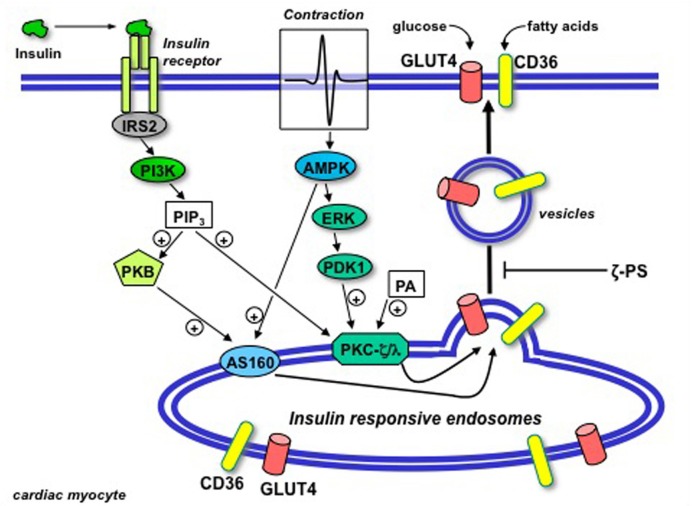
**Schematic presentation of the putative permissive role of atypical protein kinase C (isoforms ζ/λ) in GLUT4 and CD36 translocation in the heart.** Upon binding to its receptor at the cell surface, insulin sequentially activates insulin-receptor substrate 2 (IRS2) and PI3K, resulting in the production of PIP_3_. Beyond PI3K, the insulin signal is split into two parallel branches. PIP_3_ binds to both Akt/PKB and PKC-ζ/λ. In case of Akt/PKB this results in the activation and subsequent phosphorylation of AS160. In case of PKC-ζ/λ this does not result in further activation because the basally high levels of phosphatidic acid (PA) in the heart impose PKC-ζ/λ to be already bound to endosomal membranes in the absence of insulin treatment. Hence, PKC-ζ/λ maintains the endosomal membranes in a pre-active state, waiting for the insulin signal through activation of Akt/PKB and AS160 to complete the final stages in the budding of GLUT4- and CD36-containing vesicles for translocation to the sarcolemma. Upon contraction-induced activation of AMP-activated protein kinase (AMPK), a signaling cascade also involving ERK and PDK1, similarly will activate AS160 but not further activate PKC-ζ/λ. In agreement with the dependence of insulin-stimulated GLUT4- and CD36 translocation of PKC-ζ/λ, these processes will be subjected to inhibition by myristoylated PKC-ζ pseudosubstrate (ζ-PS). (Modified from Luiken et al., 2009).

In the present study we have shown that atypical PKCs are necessary but not rate-limiting in the regulation of cardiac substrate uptake (summarized in Figure 4). It should be stressed that this conclusion may be tissue-specific. Namely, as mentioned in the “Introduction,” in PKC-λ-deficient skeletal muscle insulin-stimulated GLUT4 translocation and glucose uptake were largely abolished (Farese et al., 2007). Yet, in PKC-λ deficient heart insulin-stimulated glucose uptake was fully normal, as were AMPK-mediated glucose uptake and insulin-stimulated and AMPK-mediated fatty acid uptake (present study). As a result, atypical PKCs appear to be necessary for the regulation of substrate uptake in both heart and skeletal muscle. However, only in the heart is there an overcapacity of atypical PKC isoforms in relation to insulin/oligomycin-stimulated glucose and fatty acid uptake, while in skeletal muscle the genetic ablation of PKC-λ is apparently rate-limiting for insulin-stimulated glucose uptake. Since the expression of PKC-λ and PKC-ζ is comparable between heart and muscle (Farese et al., 2007, and own data, not shown) it is difficult to explain the differential involvement of atypical PKCs in the regulation of glucose uptake. Since Farese et al. (2007) did not investigate the regulation of fatty acid uptake in PKC-λ-deficient skeletal muscle it cannot be deduced whether insulin-stimulated or AMPK-mediated fatty acid uptake would be completely blocked in this tissue by genetic PKC-λ ablation. Thus, whether there is tissue-specificity with respect to the involvement of atypical PKCs in regulating fatty acid uptake remains unknown.

We can only speculate as to the functional significance for an overcapacity of atypical PKCs in the relation to regulation of cardiac substrate uptake. However, it should be noted that in the heart atypical PKCs are involved in various other biological functions besides regulation of substrate uptake, such as the regulation of cell growth, polarity, and apoptosis (Shizukuda and Buttrick, 2002; Liu et al., 2006). It is possible that these processes require a much greater capacity of atypical PKCs than is needed for the regulation of substrate uptake.

Finally, in the healthy heart there is an optimal balance between glucose and fatty acid uptake in order to sustain proper mechanical function. In various cardiac diseases this substrate balance is disturbed, which could result in impaired functioning. Therapies aimed at normalization of cardiac substrate metabolism through selective translocation of either GLUT4 or CD36 have been proposed to protect the diseased heart against loss of function (Glatz et al., 2006). Therefore, it became of interest to search for signaling and trafficking proteins specifically dedicated to either GLUT4 or CD36 translocation, because these proteins would offer attractive targets to modulate substrate preference. However, atypical PKCs would not provide such target. First, atypical PKCs are similarly involved in the regulation of glucose uptake and fatty acid uptake by the heart, and therefore these PKC isoforms lack the ability to discriminate between both processes. Second, since PKCs do not present a rate-limiting step, a potential pharmacological activation or partial inhibition should have little, if any, effect on cardiac glucose and fatty acid fluxes.

## Acknowledgments and funding

This study was supported by the Netherlands Organisation for Health Research and Development (ZonMw grant 912-04-075), the European Community (Integrated Project LSHM-CT-2004-005272, Exgenesis), the Danish Medical Research Council, the Lundbeck Foundation, the Danish Ministry of Food, the Heart and Stroke Foundation of Ontario and the Natural Siences and Engineering Research Council of Canada (NSERC). Arend Bonen is the Canada Research Chair in Metabolism and Health.

### Conflict of interest statement

The authors declare that the research was conducted in the absence of any commercial or financial relationships that could be construed as a potential conflict of interest.

## Abbreviations

AMPK: AMP-activated protein kinase
aPKC: atypical protein kinase C
AS160: Akt substrate 160
CD36: fatty acid translocase CD36
GAPDH: glyceraldehydes-3-phosphate dehydrogenase
GLUT4: glucose transporter 4
PKC: protein kinase C
VAMP2: vesicle-associated membrane protein-2.
